# Tumor Homogeneity between Primary and Metastatic Sites for BRAF Status in Metastatic Melanoma Determined by Immunohistochemical and Molecular Testing

**DOI:** 10.1371/journal.pone.0070826

**Published:** 2013-08-20

**Authors:** Lucile Boursault, Véronique Haddad, Béatrice Vergier, David Cappellen, Severine Verdon, Jean-Pierre Bellocq, Thomas Jouary, Jean-Philippe Merlio

**Affiliations:** 1 Department of Dermatology, CHU Bordeaux, Bordeaux, France; 2 Tumor Bank and Tumor Biology Laboratory, CHU Bordeaux, Pessac, France; 3 Department of Pathology, CHU Bordeaux, Pessac, France; 4 Department of Pathology, CHU Strasbourg, Strasbourg, France; Faculdade de Medicina, Universidade de São Paulo, Brazil

## Abstract

BRAF inhibitors have demonstrated improvement of overall survival in patients with metastatic melanoma and BRAF^V600^ mutations. In order to evaluate BRAF tumor heterogeneity between primary and metastatic site, we have evaluated the performance of immunohistochemistry (IHC) with an anti-BRAF^V600E^ antibody in both localization by comparison with high resolution melting analysis followed by Sanger sequencing in a parallel blinded study. A total of 230 samples distributed as primary melanoma (n = 88) and different types of metastatic samples (n = 142) were studied in 99 patients with advanced or metastatic melanoma (stage III or IV). The prevalence of each *BRAF* mutation was c.1799T>A, *BRAF^V600E^* (45.2%), c.1799_1800TG>AA, *BRAF^V600E2^* (3.0%), c.1798_1799GT>AA, *BRAF^V600K^* (3.0%), c.1801 A>G, *BRAF^K601E^* (1.3%), c.1789_1790CT>TC, *BRAF^L597S^* (0.4%), c.1780G>A, *BRAF^D594N^* (0.9%) respectively. IHC was positive in 109/112 samples harboring BRAF^V600E/E2^ mutations and negative in other cases. The cytoplasmic staining was either strongly positive in tumor cells of BRAF^V600E^ mutated cases. It appeared strong brown, different from the vesicular grey cytoplasmic pigmentation of melanophages. Concordance between the two techniques was 96.4%. Sensitivity of IHC for detecting the BRAF^V600E/E2^ mutations was 97.3%, while specificity was 100%. Both our IHC and molecular study demonstrated homogeneity between primary and metastatic sites for BRAF status in melanoma. This study also provides evidence that IHC may be a cost-effective first-line method for BRAF^V600E^ detection. Thereafter, molecular techniques should be used in negative, ambiguous or non-contributive cases.

## Introduction

With approximately 20.000 annual deaths in Europe and an estimated median overall survival of 7 months, metastatic melanoma (MM) is the most aggressive type of skin cancer [1]. Melanoma is currently the 11th and 9th most common cancer in European women and men, respectively [2]. Melanoma incidence has risen dramatically for at least 30 years, increasing faster than any other solid tumor [3]. Until 2011, only two therapies for metastatic melanoma were Federal Drug Administration (FDA)-approved, dacarbazine and high dose interleukin 2 without demonstrated benefit in median overall survival [4] [5] [6]. Clinical, pathological and epidemiological data indicate that melanoma is composed of distinct biological subtypes supported by key molecular signaling events underlying the pathogenesis of melanoma [7] [8]. Activating mutations of the *BRAF* oncogene have been reported in 33% to 47% of primary melanomas and 41% to 55% of MM [9]. These *BRAF* mutations constitutively activate BRAF and its downstream signaling of the Mitogen-Activated Protein -kinase pathway promoting proliferation, survival and spreading of tumor cells [10] [11] [12] [13]. A quite similar rate of *BRAF* activating mutations has been also found in melanocytic nevi [14] [15]. The highest rate of *BRAF* mutation was observed in non-chronic sun-induced damage melanoma but almost all histological subtypes of melanoma may exhibit such mutation making it an attractive candidate for targeted therapy at advanced or metastatic stages [7] [16]. Furthermore, up to ninety percent of the reported *BRAF* mutations consists in a substitution of the valine residue at amino acid position 600 to glutamic acid, further referred as BRAF c.1799 T>A (BRAF^V600E^) mutation [10]. The same protein mutation may rarely results from a double nucleotide substitution (c.1799_1800TG>AA), sometimes referred as the BRAF^V600E2^ variant. The development of RAF inhibitors such as vemurafenib and dabrafenib targeting BRAF^V600E^ in melanoma cells has revolutionized the treatment of metastatic melanoma with improved progression-free and overall survival when compared with dacarbazine [17] [12] [18]. Patients with melanoma bearing other BRAF^V600^ mutations may also respond to BRAF inhibitors or/and MEK inhibitors[17] [19] [20] [21] [22] [23]. Therefore, the rapid screening for *BRAF^V600^* mutations in patients with advanced or metastatic melanoma recently became mandatory. Several DNA-based methods are currently available to determine *BRAF* status, with different sensitivities, specificities and costs [24] [25] [26] [27]. Recently, a monoclonal antibody, named VE1, specific for the BRAF^V600E^ protein has been generated [28]. To date, several studies have demonstrated a high sensitivity and specificity of immunohistochemistry (IHC) for the detection of BRAF^V600E^ mutation in MM patients by comparison with molecular techniques [24] [29] [27] [30] [31] [32]. So far, these immunohistochemical studies have not evaluated inter-tumor heterogeneity between primary and metastatic sites. Of note, a large scale study of 99 patients recently showed a 15% rate of discrepancies between primary and metastatic sites for *BRAF*/*NRAS* mutation [33]. Inter and intra-tumor genetic heterogeneity for *BRAF* status was also suggested by microdissection technique followed by molecular analysis supporting the need for testing multiple sites in a single patient or to use blood-based mutation-detection method [34].

We designed a large study of primary and metastatic melanoma samples (n = 230) in order to test in a blind manner the respective performance of IHC and molecular detection techniques for the BRAF^V600E^ mutation. By comparing primary and metastatic sites, we studied with both techniques whether *BRAF* status determination of a single sample may impact the eligibility of patients for BRAF inhibitors. As molecular techniques may also detect other *BRAF* mutations than *BRAF^V600E^*, we finally proposed a decision algorithm for the choice and the place of the detection techniques based on our results.

## Materials and Methods

### 1. Patients

One hundred and seventeen patients have been consecutively referred to our dermatology unit for MM between January 2007 and May 2012. Selection criteria included availability of tumor tissue from primary melanoma and metastasis, and pathologically confirmed stage IIIb, IIIc or IV on American Joint Committee on Cancer [34].

Clinical and follow-up data were reviewed and 18 patients were excluded for unavailability of paired melanoma samples (n = 13) or inappropriate fixation of material (Bouin's fluid) (n = 5). The cohort finally consisted of 99 patients who contributed a total of 142 metastatic tumors and 88 primary tumor specimens for analysis. Among the 99 patients, 51 (51.5%) were stage III and 48 (48.5%) were stage IV.

According to the French Public Health and Bioethical Law, our study was considered by our research direction lawyer as a non-interventional study without need for ethics committee approval (Article L1121-1 and Article R1121-3). Accordingly, all patients received from Dr. T. Jouary oral and written information for research on their material with possibility to refuse the use of their data and material. Moreover, results of the supplemental immunohistochemical detection of BRAFV600E were collected blindly and anonymously while molecular analyses were performed as standard testing for patients' management and diagnosis.

### 2. Samples

Tumor specimens included 88 primary melanomas, 81 lymph node metastases, 45 skin metastases, 14 visceral metastases (lung n = 6, liver n = 4, spleen n = 1, parotid n = 1, stomach n = 1 and pancreas n = 1), cerebral metastasis (n = 1), bones metastasis (n = 1) (Table 1). All samples were formaldehyde-fixed and paraffin-embedded (FFPE). All primary melanoma samples referred to our hospital were reviewed by a dermatopathologist (BV) to assess primary melanoma diagnosis and histological subtype. All secondary/metastatic samples were also evaluated by additional immunostaining for MelanA, HMB45 and S100 protein (all from DAKO, Courtaboeuf, France), to confirm the melanoma origin. Finally, 230 samples were analyzed in parallel for *BRAF* exon 15 mutations and BRAF^V600E^ protein expression.

**Table 1:** Comparative data for BRAF status evaluation in primary and metastastics sites.

		BRAF status			BRAF immunostaining		
Sample	N° samples	results	No	%	results	No	%
**Primary tumor**	88	wt	42	52,27	negative	41	46,59
					NI	1	1,14
		V600E	39	43,18	positive	38	43,18
					NI	1	1,14
		V600E2	4	3,41	positive	4	4,55
					NI	0	0,00
		V600K	3	3,41	negative	3	3,41
					NI	0	0,00
**All metastatic sites**	142	wt	63	44,37	negative	63	44,37
					NI	0	0,00
		V600E	65	46,76	positive	63	44,37
					NC	2	1,41
		V600E2	4	2,88	positive	4	2,82
		V600K	4	2,88	negative	3	2,11
					NI	1	0,70
		K601E	3	2,16	negative	3	2,11
		L597S	1	0,72	negative	1	0,70
		D594N	2	1,44	negative	2	1,41
Lymph node metastasis	81	wt	33	40,74	negative	33	40,74
					NI	0	0,00
		V600E	40	49,38	positive	40	49,38
					NI	0	0,00
		V600E2	3	3,70	positive	3	3,70
					NI	0	0,00
		V600K	3	3,70	negative	2	2,47
					NI	1	1,23
		K601E	1	1,23	negative	1	1,23
		L597S	1	1,23	negative	1	1,23
		D594N	0	0,00	negative	1	0,00
Skin metastases	45	wt	22	50,00	negative	22	48,89
		V600E	19	43,18	positive	17	37,78
					NC	2	4,44
		V600E2	1	2,27	positive	1	2,22
		K601E	2	4,55	negative	2	4,44
		L597S	0	0,00		0	0,00
		D594N	1	2,27	negative	1	0,00
Bone metastases	1	wt	1	100,00	negative	1	100,00
Brain metastases		V600E	1	100,00	positive	1	100,00
Visceral metastases	14						
Liver	4	wt	3	75,00	negative	4	100,00
		V600K	1	25,00			
Lung	6	wt	2	33,33	negative	2	33,33
		V600E	4	66,67	positive	4	66,67
Spleen	1	wt	1	100,00	negative	1	100,00
Parotid	1	wt	1	100,00	negative	1	100,00
Pancreas	1	V600E	1	100,00	positive	1	100,00
Stomach	1	D594N	1	100,00	negative	1	100,00
**total**	**230**						

NI: non interpretable (ambiguous case). NC: non contributive.

### 3. Immunohistochemistry for BRAF^V600E^ protein

Anti-BRAF^V600E^ immunostaining was performed on 4 µm thick tissue sections of the same FFPE material used for mutation testing, using the monoclonal mouse antibody VE1 [28] provided by Spring Biosciences (Pleasanton, USA). Immunostaining was performed on Ventana® Benchmark XT immunostainer (Roche Diagnostics, Meylan, France) at University Hospital of Strasbourg using the Ventana Optiview diaminobenzidine immunohistochemistry detection kit, following the manufacturer's procedure (CC1© pretreatment for 64 min, antibody dilution at 1∶100, incubation at 37°C for 16 min, OptiView detection kit© with OptiView Amplification©). A positive control was included in each IHC round. All slides were freshly cut less than two weeks before IHC technique and stored at 4°C. A first set of ten slides (with four mutated BRAF^V600E^ cases) was analyzed as a training set by both reviewers in order to set up common interpretation criteria of positive cases and to identify possible artifacts. Then, all slides were evaluated independently by 2 observers (BV and JPM) blinded to clinical and molecular data. Immunostaining was primarily interpreted as positive or negative according to Capper et al. [28]. The VE1 antibody staining was scored as positive when the tumors cells showed a clear cytoplasmic staining. The VE1 antibody was scored as negative when there was no staining or only nuclear dot staining, weak staining of single interspersed cells, staining of monocytes/macrophages. Cases were scored as ambiguous if immunostaining could not be scored as positive or negative. Cases scored initially as ambiguous or non-interpretable were retested.

### 4. Mutation analysis

After selection of the relevant paraffin blocks on hematein-eosin stained section, genomic DNA was extracted from 5 sections of FFPE material (5 µM thickness each) using the QIAamp DNA FFPE extraction kit and the QIAcube automated DNA extraction machine (Qiagen, Courtaboeuf, France). All experimental procedures were performed according to the manufacturer's protocol. In 5 cases with an estimated percentage of tumor cell content of less than 10%, the tumor area was macrodissected using a 20-gauge 1.5-inch blunt needle (Sigma Aldrich Réf: Z708801-25EA ) that was pushed through the entire block prior to DNA extraction as previously described [35]. Thirty ng of DNA samples were subjected in duplicate to high resolution melting (HRM) analysis on a LC480 device (Roche Diagnostics), using primers flanking codon 600 in the *BRAF* gene (5′TTCATGAAGACCTCACAGTAAAAA3′ and 5′CCACAAAATGGATCCAGACA3′). The HRM protocol consisted of an initial denaturation of 95°C for 10 minutes, followed by 55 cycles of amplification consisting of denaturation at 95°C for 15 seconds, annealing at 60°C for 25 seconds, and extension 72°C for 15 seconds. Melting was performed with a denaturation step at 95°C for 1 minute, followed by an annealing step at 40°C for 1 minute and a melt from 70°C to 95°C at a ramp rate of 0.03°C/second with 25 acquisitions per degree Celsius. The Roche analysis software version 1.5.0.SP3 (Roche Diagnostics) was used to detect variant sequences and the settings were optimized to achieve maximum sensitivity. These primers amplify a 107 bp long fragment allowing the detection of exon 15 sequence variation of the *BRAF* gene between nucleotides c.1742 and c.1838 by comparison with the reference sequence NM_00433.4, corresponding to codon 581 to 612. All amplified fragments with a variant profile (≥3 Standard Deviation) or Temperature melting (Tm) measurement differing from wild type control were subjected to DNA Sanger sequencing of both strands. Sensitivity testing experiments were performed using the Horizon Diagnostics control slides of FFPE cell lines block with defined ratios of mutant alleles (1 wild type sample, 1 at 50%, 1 at 3.5%), enabling the quality control of both assay sensitivity and DNA extraction (Horizon Discovery, Cambridge, UK). We also performed dilution experiments of the 50% mutated DNA into the wild-type DNA.

### 5. Statistical analysis

Analyses were performed with Prism 6 (Graph Pad Software). Sensitivity and specificity were performed using Fischer's exact test for 95% confidence interval. Correlation between the two techniques was performed with an unpaired t-test (Spearman's test, 95% confidence interval). BRAF status versus type of melanoma site (primary melanoma/metastasis) was studied with paired t-test (Spearman's test, 95% confidence interval).

## Results

### Patients and tumors samples

Our study included 99 patients with MM and 230 samples. BRAF status was determined in a blinded manner by molecular and immunohistochemical techniques (Table 1). The presence of *BRAF* mutation was also analyzed according to the histological subtype of primary melanoma (Table 2).

**Table 2:** *BRAF* status according to histological subtype of the primary melanoma site.

primary	patients (nb)	%
SSM	56	63,64
V600E	27	48,21
V600E2	2	3,57
V600K	3	5,36
K601E	0	0,00
wt	24	42,86
nodular	20	22,73
V600E	10	50,00
V600E2	2	10,00
wt	8	40,00
lentiginous	1	1,14
wt	1	100,00
mucous	3	3,41
wt	3	100,00
acro-lentiginous	4	4,55
V600E	1	25,00
wt	3	75,00
fusiform cells	3	3,41
wt	3	100,00
melanoma on naevus	1	1,14
V600E	1	100,00
total	88	

SSM: superficial spreading melanoma; Wt: wild type.

### *BRAF* mutation detection

Genetic analyses were interpretable in all 230 samples (100%). This resulted in the detection of a *BRAF* mutation in 125/230 samples (54.3%), including 46/88 (54.5%) primary tumors and 79/142 (55.6%) metastatic tumors. One hundred and four samples were found to harbor the *BRAF* c.1799T>A (BRAF^V600E^) mutation (45.2%), 8 were c.1799_1800TG>AA (BRAF^V600E2^) (3.5%), 7 were c.1798_1799GT>AA (BRAF^V600K^) (3.0%) and 3 c.1801 A>G (BRAF^K601E^) (1.3%). Moreover, c.1789_1790CT>TC (BRAF^L597S^) and c.1780G>A (BRAF^D594N^) *BRAF* mutations were detected in one and two samples (0.4% and 0.9%), respectively (Table 1). Dilution experiments showed that our HRM technique had a threshold at 3.5% of *BRAF^V600E^* mutated allele. Further identification of the mutation by Sanger sequencing in such samples required 5% of mutated allele (data not shown). None sample provided discordant data between the HRM profile and the subsequent Sanger sequencing as all samples contained more than 10% of tumor cells, except the 5 cases that required macro-dissection to enrich tumor cell content above this threshold.

### Immunohistochemistry

**Figure 1:**
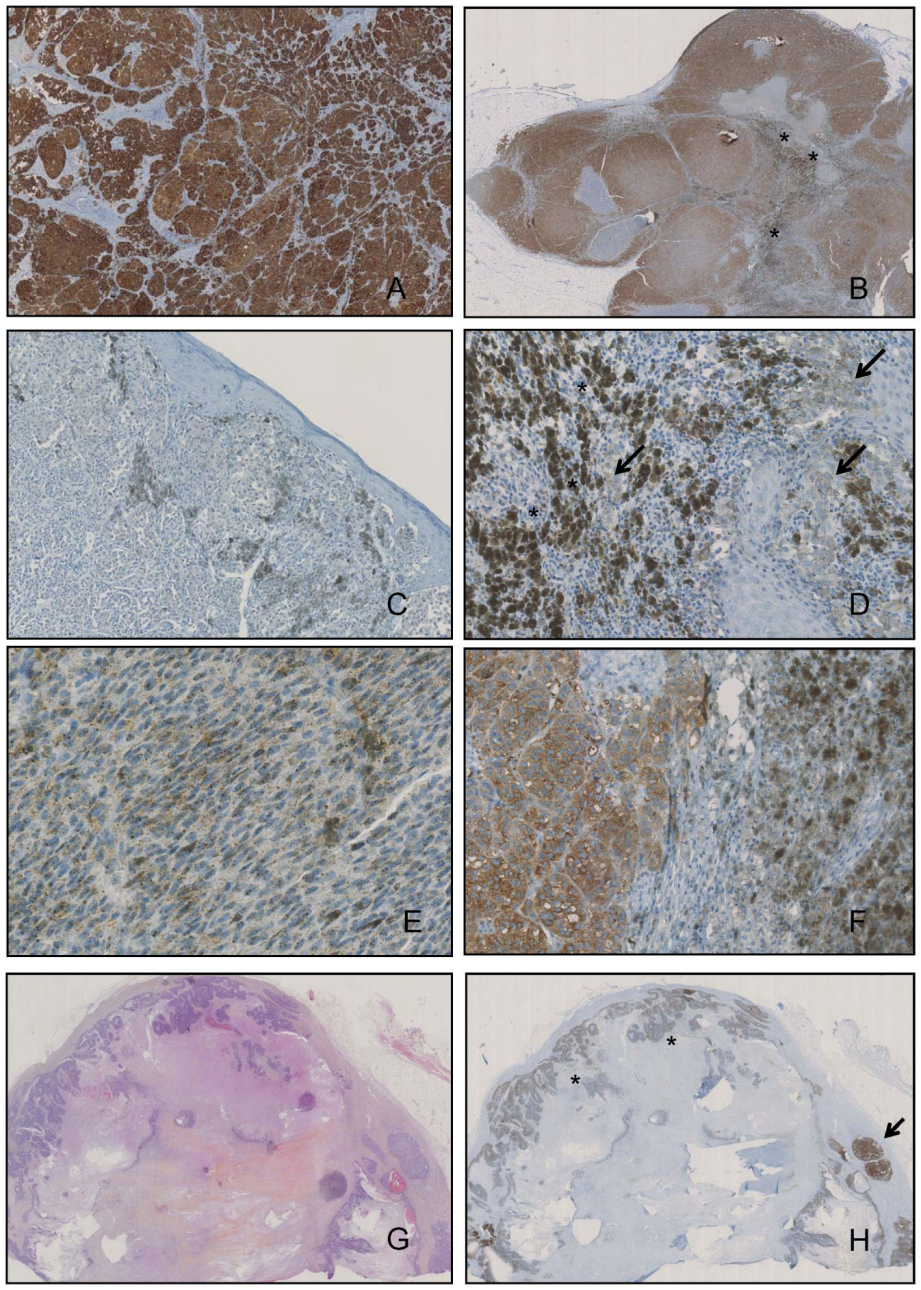
VE1 immunostaining for BRAF p.V600E in positive, negative and ambiguous cases. A. Positive case showing diffuse dark brown cytoplasmic immunostaining. Immunoperoxidase staining ×50, magnification. B. Metastatic lymph node showing positive staining with homogeneity between tumor areas. Note the presence of melanophages with gray-green cytoplasmic dots (asterisks) in sinuses. Immunoperoxidase staining ×10, magnification. C. Primary cutaneous sample of a case without BRAF mutation. Note the absence of staining of tumor cells and the presence of gray-green pigment in melanophages. Immunoperoxidase staining, ×250 magnification. D. Ambiguous staining in a primary melanoma without BRAF p.V600E mutation as detected by molecular techniques. Presence of a faint staining of tumor cells (arrows) contrasting with the intense pigmentation of melanophages (asterisks). In such cases (3 in our study) it was not possible to assess whether tumor cells were immunostained or pigmented. Immunoperoxidase staining, ×400 magnification. E. Negative immunostaining case for BRAF p.V600E in a primary melanoma showing artifacts with diffuse and nuclear small brown dots differing from the cytoplasmic staining of the positive cases. Immunoperoxidase staining, ×400 magnification. F. Metastatic lymph node with positive brown cytoplasmic staining of tumor cells and gray-green pigmentation of melanophages. Immunoperoxidase staining ×400 magnification. G. Metastatic lymph node involved by melanoma. Extensive necrosis with peripheral tumor areas. Hematein-eosin staining, ×10 magnification. H. Same sample than in E. Adjacent section immunostained with the VE1 antibody showing positive tumor area (arrow) while necrosis remains negative (asterisks). Immunoperoxidase staining ×10 magnification.

The staining obtained with VE1 antibody was assessed in the 230 samples. Samples were scored as positive with a moderate to strong brown staining of more than 90% of tumor cells (Fig. 1A and 1B) or negative (Fig. 1C). Two samples (0.9%) were non contributive as the slide did not contain any tumor cell. In 3 samples (1.3%), an ambiguous pattern was observed with a faint and equivocal brown staining of tumor cells (Fig. 1D). This ambiguous pattern differed with the description of a background staining, defined as diffuse dots covering also the nuclei (Fig. 1E). Moreover in pigmented samples, melanin accumulation appeared like green-gray cytoplasmic dots observed mostly in melanophages or sometimes in tumor cells (Figure 1B, 1C, 1D and 1F).

Based on the above criteria, BRAF^V600E^ expression was observed in 109/230 tumor samples (47.4%). The strong cytoplasmic staining for BRAF^V600E^ was almost homogeneous throughout tumor areas (Fig. 1B) and cells. In some metastatic lymph nodes, necrotic areas and adjacent tumor cells showed absent or weak staining (Fig. 1G and 1H). Difference in staining intensity between tumor areas was rarely observed and linked to the presence of necrosis, fixation artifacts or sometimes difference in morphological differentiation of positive tumor cells stained for BRAF^V600E^. Concordance for the VE1 immunostaining analyses between both observers was complete (100%), as all positive and negative cases were scored similarly. Both observers also agreed for the 3 ambiguous cases that were independently all scored neither positive nor negative (Fig. 1D).

### Correlation between immunohistochemical and molecular data

Correlation analyses were performed both for BRAF^V600E^ and for other *BRAF* mutations (*BRAF* ^mt^) which are relevant for patients who may benefit from BRAF or MEK inhibitors molecules. The 109 BRAF^V600E^ immunopositive samples were all detected also by molecular analysis either as c.1799T>A (BRAF^V600E^) (n = 101) or c.1799_1800TG>AA (BRAF^V600E2^) (n = 8) mutations corresponding to a perfect match between the two techniques in informative samples. As expected, no immunostaining difference was observed between *BRAF^V600E^* and *BRAF^V600E2^* mutations, as they encode for the same BRAF^V600E^ protein. A *BRAF^V600E^* mutation was identified by molecular techniques in two samples in which no more tumor cell was present in the immunostained slides and in one out of the 3 cases with ambiguous immunostaining. Conversely, among the 112 samples with BRAF^V600E^ mutation identified by molecular techniques, immunostaining was scored positive in 109 (97.3%). No false positive results were observed for IHC. Thus, IHC sensitivity for BRAF^V600E^ detection was 97.3% (CI: 92.37–99.44) and specificity was 100% (CI: 96.55–100) with a positive predictive value of 100% (CI: 96.67–100), and a negative predictive value of 97.2% (CI: 92.10–99.42). Concordance between the 2 techniques was 96.4% (CI: 95.26–97.24).

Among the 116 IHC negative cases, molecular analysis showed a *BRAF* wild-type (*BRAF^wt^*) status in 104 samples (89.6%), a BRAF^V600K^ mutation in 6 (5.2%), a BRAF^K601E^ mutation in 3, (2.6%), a BRAF^L597S^ mutation in one (0.9%) and a BRAF^D594N^ in two (1.7%). Among the 3 ambiguous IHC samples, *BRAF* sequencing showed a BRAF^V600E^ mutation (n = 1) as described above, a BRAF^V600K^ mutation (n = 1) and a wild type status (n = 1). (Table 1)

Therefore, for BRAF^V600^ mutation, IHC sensitivity was 91.6% (CI: 85.09–95.90) and specificity was 100% (CI: 96.84–100%). Finally, mutations BRAF^V600K^, BRAF^K601E^, BRAF^L597S^ and BRAF^D594S^ were not detected by IHC. Concordance between the 2 techniques was 87.8% (84.29–90.50) for BRAF mutation detection, and 90.6% (CI: 87.89–92.78) for specific *BRAF* V600 detection.

### Concordance between paired-samples in patients

Of the 99 patients, genetic analysis revealed a *BRAF* mutation in 52 (52.5%). Forty-three patients harbored *BRAF^V600E^* mutation (43.4%), 3 BRAF^V600E2^ (3.0%), 3 BRAF^V600K^ (3.0%), 1 BRAF^K601E^ (1.0%), 1 BRAF^L597S^ (1.0%) and 1 BRAF^D594N^ (1.0%). Ninety-five patients showed perfect concordance of the *BRAF* status between two or more samples (96.0%). Eighty-eight patients had paired-samples composed of one sample from the primary tumor and one sample from a metastatic lesion. The *BRAF* status was concordant between the primary and metastatic samples for 84 patients whatever the site of metastasis (90.9%) (CI: 86.29–94.05). For patients with more than one metastatic sample (n = 37), *BRAF* status was concordant (100%) between all metastatic sites for each patient.

Discordant results for *BRAF* status were observed in 4 patients out of 88 (4.5%). Interestingly, this discordance was always noted for paired-samples between a primary and a metastatic sample. One patient had a *BRAF* mutation in the primary melanoma and not in the two metastatic sites (bone and liver); three patients had a *BRAF* mutation in the metastatic tumors and not in primary tumors. The metastatic sites were lymph node in 2 cases and stomach and skin in 1.

## Discussion

Detection of *BRAF* ^V600^ mutation is now a pivotal and decisional factor in the stratification and the treatment strategy of patients with MM [36] [12]. The *BRAF^V600E^* accounts for approximately 90% of all *BRAF* mutations in MM. Its detection has been initially achieved using several different molecular techniques including allele-specific real-time quantitative polymerase chain reaction (q-PCR), pyrosequencing, high resolution melting (HRM) and/or Sanger sequencing (for review see Curry JL et al 2012 [26]).

More recently, IHC with a new antibody directed against the BRAF protein has challenged molecular techniques for such predictive testing, as shown by the designers of this new tool [24] [29] [27] [31]. Our independent study of 230 samples from 99 patients extends their findings and underlines the need for standardization of interpretation criteria.

By comparison with molecular data, IHC detection of BRAF^V600E^ achieved 97.3% sensitivity and 100% specificity, as well as 100% inter-observers agreement. Although the staining for BRAF^V600E^ was cytoplasmic, intense and brown, some patterns have to be kept in mind using this new tool. As the staining of melanophages was previously described using a similar chromogen [27] [28] we observed that the green-gray melanin pigmentation of tumor cells or melanophages appeared different from the brown cytoplasmic staining for BRAF^V600E^. An ambiguous staining of tumor cells was only observed in 3 samples (n = 3) and was also reported in another study using Fast Red as a chromogen [24]. Late fixation, overfixation or even per-operative traumatisms like surgical coagulation may also account for such pitfalls [28] [37]. Moreover, we also observed that epitope antigenicity seemed to be impaired by necrosis and in areas adjacent to necrosis that could mislead to a false negative result. Using the very high-sensitive Optiview amplification system, we have not observed intratumoral heterogeneity with positive and negative cell clusters within the same tumor sample. Staining heterogeneity was recently reported in primary and metastatic samples but neither staining heterogeneity nor intensity scoring were found relevant for predicting outcome in patients treated by BRAF inhibitors [32] [38]. Primary SSM developed on a preexisting nevus was also reported to exhibit stronger staining intensity for BRAF^V600E^ than the benign component [32]. Altogether, such staining variations may have several causes and were not shown to match with difference in BRAF genetic status.

Some studies have supported the need for using highly sensitive molecular techniques in MM such as pyrosequencing and allele specific-q-PCR, that was supported by testing their sensitivity on serial dilutions of DNA extracted from a cell line carrying a heterozygous *BRAF* c.1799T>A (V600E) mutation [24] [25] [26] [39] [40]. Our data does not support such findings in daily practice as we observed almost a perfect match between IHC with VE1 antibody and molecular detection by HRM followed by Sanger sequencing of the c.1799T>A *BRAF^V600E^* and the c.1799TG>AA (*BRAF* ^V600E2^). Here, such an excellent performance of the HRM technique could be explained by the primers design, their position from the mutation site, the short length of amplicons and the use of 55 cycles of amplification prior to melting curves analysis and Tm determination as these parameters are critical [41]. In fact, more than 10% of tumor cells, which is a common feature in metastatic melanoma samples, were the minimal required threshold to obtain consensus among five mutation detection assays [25]. Therefore in 3 samples among 7 with less than 10% of tumor cells, macrodissection of the FFPE tumor area for the detection of a *BRAF^V600E^* mutation was used and matched our IHC results.

In addition, HRM technique allowed the detection of less common *BRAF^V600^* mutations in 7 cases as well as the detection of *BRAF* mutations at other positions than codon 600 in 6 cases. The less common *BRAF* ^V600^ mutations, i.e. V600D and K also reported by others [24] [25] [33] were found to represent 3.0% of our patients. Indeed, the *BRAF^V600K^* mutation was shown to predict response to BRAF inhibitors in several reports while BRAF mutations out of V600 are not so far eligible to *BRAF* inhibitors [12] [19] [20] [42]. Dalhman *et al.* [43] reported that one patient with the *BRAF^L597S^* mutation displayed clinical response to MEK inhibitors underlining the need for a molecular testing. The COBAS 4800 *BRAF* ^V600^ mutation test was developed by Roche on the principle of allele specific q-PCR in order to achieve standardization, specificity and sensitivity [26] [44]. The COBAS assay, with qualitative standardized results (mutation detected, mutation not detected or invalid result), does not distinguish the *BRAF^V600E^* from other *BRAF^V600^* mutations. The COBAS assay, which is so far the only FDA-approved test for selecting patients, should be therefore tested along IHC for BRAF^V600E^ detection.

Using molecular techniques, several studies have reported intra-tumor heterogeneity for BRAF status in melanoma [45] [46]. Using laser microdissection and mutation specific Snapshot assay, Yancovitz *et al.* [34], have found that a substantial proportion of individual tumor specimens contained a mixture of BRAF mutant and wild type melanoma cells. Such intra-tumoral heterogeneity would account for discrepancies between molecular techniques in detecting *BRAF* mutation according to their sensitivity threshold supporting the use of very sensitive techniques [25]. In accordance with other IHC studies [27] [29] [31] [24], we observed homogeneous staining of tumor cells that seems to rule out the hypothesis of intra-tumoral heterogeneity. Our results also differ from studies reporting different *BRAF* status between different metastases according to the MM site [33] [34] [47]. We have found 100% stability for *BRAF* status between different metastases in the same patient (n = 37) and observed an inter-tumor concordance of 90.9% between primary and paired-metastatic sites (n = 88), with only 4 discordant patients (4.0% patients). In one patient, clinical history supported the emergence of a second melanoma with a different *BRAF* status. This patient received dacarbazine for MM discovered on bones metastasis during several months because of *BRAF* wild type status (both by IHC and molecular techniques) while the primary melanoma was unknown. Five months later, a pigmented lesion was discovered, exhibiting histological features of primary melanoma, which was found to display the BRAF^V600E^ mutation. This lesion was probably a second melanoma, with a different *BRAF* status. In two other cases, the primary melanoma had a *BRAF* ^wt^ status and a very rare mutation was discovered in metastases (BRAF^L597S^ and BRAF^D594N^), suggesting that they could have been acquired during tumor progression. Conversely, the common *BRAF^V600E^* mutation and the other *BRAF^V600^* mutations were found homogeneous in our patients as also reported for *BRAF^V600E^* in patients under treatment by BRAF inhibitors [48]. Finally, only one case with wild type primary melanoma and *BRAF^V600E^* metastatic lymph node was identified by both methods and remains unique in our study.

## Conclusions

In this large series, IHC with VE1 antibody appeared as a sensitive and specific tool for the detection of BRAF^V600E^ protein in FFPE tissue using appropriate interpretation criteria. As *BRAF* ^V600E^ staining was found both homogeneous between tumor cells and conserved between primary and metastatic samples, the initial testing could be performed on any available tumor tissue, issued from either primary or secondary lesions. Staining for BRAF^V600E^ will give a global overview of the patient status for treatment decision. By comparison, the use of very sensitive molecular techniques that would detect a minor BRAF-mutated subclone in a predominantly wild-type tumor may not be clinically relevant as BRAF inhibitors may have opposite signaling effects in cells with mutated or wild-type BRAF [49] [50]. Then, molecular testing would only be required for ambiguous or VE1-negative cases in order to either confirm IHC result or to detect a less common *BRAF* mutation (6.1% of our patients).
